# Robust and cost-saving static solid cultivation method for lipid production using the chlamydospores of *Phanerochaete chrysosporium*

**DOI:** 10.1186/s13068-019-1464-1

**Published:** 2019-05-20

**Authors:** Lei Liu, Jie Song, Yi Li, Ping Li, Hailei Wang

**Affiliations:** 1 College of Life Sciences, Henan Normal University, Xinxiang, 453007 China; 2 Advanced Environmental Biotechnology Center, Nanyang Environment and Water Research Institute, Nanyang Technological University, Singapore, 637141 Singapore

**Keywords:** *Phanerochaete chrysosporiu*m, Lipids, Solid cultivation, Chlamydospore, Cost-saving

## Abstract

**Background:**

During current submerged fermentation for microbial lipid production, the large-scale reactor operations inevitably consume substantial amounts of water and electricity for aeration, stirring, and temperature control and result in the operational costs almost exceeding the biodiesel value produced. Thus, developing a novel low-cost cultivation strategy is urgently needed by microbial lipid industry.

**Results:**

The filamentous fungus *Phanerochaete chrysosporium* can synthesize and accumulate lipids via static solid cultivation. The conversion efficiency of substrates to lipids reaches 0.277 g/g substrate after optimization of the following cultivation factors: humidity, solid medium thickness, temperature, and rotary speed. The lipids obtained by static solid cultivation differ in component and relative content from those achieved by submerged cultivation. Laser scanning confocal microscopy reveals that numerous chlamydospores filled with lipids appear during static solid cultivation, and the fungal morphological change explains why static solid cultivation is superior in lipid yield compared with submerged fermentation. The genes coding the enzymes related to fatty acid elongation and degradation are differently expressed during static solid cultivation, which presents an answer to the appearance of abundant saturated long-chain fatty acids (93.6% in total fatty acids) in chlamydospores. In addition, engineering viability and cost–benefit analysis show that the conversion of wheat bran and glucose to lipid by the fungus is efficient. More importantly, the solid cultivation incurs only a small reactor operational cost because neither cooling water nor electrical equipment, including aerator, stirrer, and the temperature control system, is used.

**Conclusions:**

This study developed a robust and cost-saving solid fermentation method without an aerator, stirrer, and temperature control system to produce microbial lipids using the chlamydospores of *P. chrysosporium*. Compared with conventional submerged fermentation, the solid cultivation strategy is promising because it diminishes most of the reactor operational costs, including water and power expenses.

**Electronic supplementary material:**

The online version of this article (10.1186/s13068-019-1464-1) contains supplementary material, which is available to authorized users.

## Background

Currently, the world economy is heavily dependent on non-renewable fossil fuels, which represent over 75% of energy needs. However, fossil fuels are depleting because of enormous consumption, and burning fossil fuels is believed to cause increased levels of greenhouse and other noxious gases in the atmosphere [1]. Biodiesel has been implemented as a major biofuel product to address the concerns of fossil fuel overconsumption; as a result, the demand for commodity lipids, including vegetable oils and animal fats, as biodiesel sources has increased [2, 3]. Lipids from microbes have been considered as an alternative feedstock for biodiesel production, considering that lipids from vegetables and animals are extremely limited resources due to scarce arable lands and valuable food resources [4]. Therefore, some oleaginous microbes, defined as microorganisms capable of producing > 20% lipid of cell dry weight, have been screened and modified to overproduce microbial lipids [5–8]. Literature surveys revealed that at least 14 genera of microalgae, 4 genera of yeast, 4 genera of bacteria, and 4 genera of fungi can accumulate lipid contents of 20–86% by dry weight [9, 10].

Lipids from microalgae have been extensively investigated as a technically viable alternative energy source because they are cultivated autotrophically by photosynthesis and heterotrophically by utilizing various organic carbon sources to accumulate intracellular lipids. However, despite its popularity, microalgal lipid has its disadvantages, including low lipid productivity, long fermentation duration, large biomass harvest consumption, and high lipid extraction cost [11, 12]. Yeast is another popular organism exploited for microbial lipid production. *Saccharomyces cerevisiae*, *Rhodosporidium toruloides*, and *Yarrowia lipolytica* have been considered as ideal hosts for lipid development, mainly because of their high fermentative capability and substantially available biological information and the availability of powerful genetic manipulation tools [13]. Nevertheless, the lipid productivities of yeast strains, including *Y. lipolytica*, remain relatively low for industrial production. Thus, extensive efforts on metabolic engineering have been exerted to improve the lipid productivity of yeast [14–17]. Besides microalgae and yeast, some reports have emerged on bacteria, such as *Mycobacterium*, *Nocardia*, *Rhodococcus*, and the engineered *Escherichia coli*, for lipid production, although many of these bacteria are not effective lipid producers. Bacteria often accumulate relatively complex and diverse lipids, including fatty alcohols, glycolipids, or phospholipids, mainly in their outer membrane; as such, bacterial lipids are extremely difficult to extract [18]. Meanwhile, oleaginous fungi such as zygomycete species, e.g., *Cunninghamella echinulata* and *Mortierella isabellina*, are also potential biolipid sources. However, their lipid yields are low, and further yield improvements are necessary for economic feasibility [13, 19, 20].

At present, fossil fuel, which serves as the dominant energy source, is still much cheaper than other sources. Biofuels have only recently entered the research and development stage despite being regarded as a revolutionary technology [21]. The production cost of microbial lipids remains prohibitively high for commercialization given the low price of diesel. How to reduce their production cost has become a bottleneck impeding the development of biodiesel industry. Generally, the production cost of lipids in industrial scale mainly accounts for the fermentation substrates, reactor operation (including equipment depreciation and water and energy consumption during reactor operation), and lipid preparation cost (including biomass harvest and lipid extraction) [22, 23]. As intracellular products, biomass harvest and lipid extraction are indispensable components of microbial lipid production. Therefore, many physical, chemical, and biotechnological methods have been developed to reduce the preparation costs. For instance, the cost of ionic liquid synthesis from industrial crude materials is only 1.85% that of the commercial reagent used for lipid extraction [24, 25]. To conserve the cost of fermentation substrates, cheaper industrial and agricultural wastes, including bagasse, corncob, and lignocellulosic biomass, were utilized as carbon sources because although glucose is a highly efficient carbon source for cell growth and lipid production, its cost approximately contributes to half of the total production cost in a typical submerged cultivation (SC) process [13, 26, 27]. Researchers have also focused on increasing the lipid yield by various methods, including strain gene modification, reactor parameter optimization, and advanced fermentation processes such as high-cell-density cultivation and continuous batch-fed cultivation, to reduce the production cost [4, 8, 28, 29]. Although lipid production costs are significantly reduced by using these strategies, there is still a long way to go before an economically viable lipid yield can be obtained because of the high and commercially unfeasible biomass cultivation cost of SC. To date, SC is the most popular cultivation process for microbial lipid production [30]. However, during microalgal cultivation, the cost of CO_2_ supply from bubbling gas constitutes approximately 50% of the biomass production cost in a raceway pond. Meanwhile, the biomass harvest cost of microalgae in open pond systems or photobioreactors accounts for 30–50% of the biodiesel production cost, which is 6–10 times higher than that of diesel oil production from petroleum [24, 31]. For the cultivation of bacteria, yeasts, and fungi, an expensive fermenter is required. Furthermore, large-scale reactor operations inevitably consume substantial amounts of water and electricity for aeration, stirring, and temperature control and result in costs that far exceed the biodiesel value produced. Thus, developing a novel low-cost cultivation strategy is urgently needed by the microbial lipid industry.

In the present work, a static solid cultivation (SSC) method was developed to cultivate *Phanerochaete chrysosporium* for lipid production. To the best of our knowledge, limited information is available on the overproduction of fungal lipids by SSC. More interestingly, compared with SC, which is extensively used for lipid production, SSC conserves water and electricity during fermenter operation because of the eliminated need for expensive stirring, aeration, and temperature control systems. Therefore, the unique cultivation strategy is promising for application in industrial lipid production. This study is projected to benefit future lipid fermentation by significantly reducing costs.

## Methods

### Chemicals and microorganism

All chemicals used in this work were of analytical grade. Nile red and Calcofluor fluorescent stain were purchased from Sigma Corporation (St. Louis, MO, USA). *P. chrysosporium* (ATCC24725) was provided by the Singapore–China JointEngineering Laboratory for Bioconversion Technology of Functional Microbes (SCJEL_btfm_), Xinxiang, China. The strain was an alcohol dehydrogenase mutant [32] developed by a mutation system based on atmospheric pressure glow discharge plasma (Siqingyuan Biotechnology Co., Ltd., Wuxi, China) and stored on a potato dextrose agar (PDA) plate at 4 °C before use.

### Components of wheat bran and corn straw

Both wheat bran and corn straw used in this work were collected from the Sijiqing Farmland in Xinxiang, Henan Province, China, and their components are shown in Table 1. The protein, starch and lipid contents in wheat bran, and water content in the solid medium were analyzed in accordance with the APHA standard methods [33]. Meanwhile, the cellulose amounts were obtained as described by Yang et al. [34], and the total sugar was determined by the Somogyi–Nelson method [35]. Before use, corn straw was chopped into small pieces (50 mesh) using a fodder grinder (Henan Ruyi Machinery Co., Ltd., China).

**Table 1:** Components of wheat bran and corn straw used in this work

Wheat bran	Content (w/w, %)	Corn straw	Content (w/w, %)
Starch^a^	60.9 ± 5.53	Cellulose	38.2 ± 5.01
Cellulose	12.4 ± 0.75	Hemicellulose	23.7 ± 1.43
Protein	16.8 ± 1.08	Lignin	12.7 ± 0.49
Lipid	4.1 ± 0.33	SS^b^	15.0 ± 0.88
Others	5.8 ± 0.39	Others	11.4 ± 0.67

^a^Based on dry weight

^b^SS: soluble substance, includes soluble saccharides, starch, and a small amount of protein

### Initial SC and SSC

*Phanerochaete chrysosporium* was grown on a PDA plate for 3 days at 30 °C before the conidia were harvested using a sterile hair brush. Then, a conidium suspension was prepared in sterile water. Initial SC was carried out as follows: Three 500 mL glass bottles each containing 200 mL of liquid medium (37.5 g/L mixture of wheat bran, corn straw and glucose [1:1:2, w/w], 1.0 g/L NH_4_Cl, 0.1 g/L CuSO_4_·5H_2_O, and 0.1 mg/L veratryl alcohol) were inoculated with 0.5 mL aliquot of conidium suspension (3.0 × 10^7^ spores/mL). The bottles were incubated in a thermostat shaker at 120 rpm and 28 °C. SSC was subsequently conducted in three 500 mL bottles and cultivated statically in a thermostatic shaker (30 °C). The components of the solid medium in each bottle were as follows: 7.5 g mixture of wheat bran, corn straw and glucose (1:1:2, w/w), 0.2 g NH_4_Cl, 0.02 g CuSO_4_·5H_2_O, and 0.02 mg veratryl alcohol. These bottles were inoculated with the cultures of *P. chrysosporium* from SC obtained on day 5 (inoculum quantity is 1.0 g/bottle, dry weight), and the dry weight of cultures was determined by drying sample at 70 °C to constant weight. The initial medium humidity was 60%. Both SC and SSC were conducted for 12 days.

### Optimization of SSC parameters by One-factor-at-a-time (OFAT)

During SSC, the cultivation parameters, including humidity, thickness of the solid medium, temperature, and rotary speed of the thermostatic shaker, were optimized. In the humidity test, the initial humidity of the solid medium was adjusted to different levels (40%, 50%, 60%, 70%, and 80%). The medium thickness and inoculum quantity were 1.8 cm and 1.0 g/bottle (dry weight), respectively. SSC was conducted at 30 °C in 500 mL bottles. In the medium thickness test, the solid medium’s thickness was controlled at 0.5, 1.0, 1.5, 2.0, 2.5, and 3.0 cm. In the temperature test, six bottles were cultivated at 20 °C, 25 °C, 30 °C, 35 °C, 40 °C, and 45 °C. In the rotary speed test, four bottles were incubated in different thermostatic shakers with rotary speeds of 50, 100, 150, and 200 rpm. Each test was repeated three times, and the optimized fermentation parameter was immediately applied to the next optimization test.

### Robust SSC

SSC of *P. chrysosporium* in glass bottles was carried out in March 1, July 5, and October 1 at room temperature in 2016 to investigate the feasibility of lipid fermentation without temperature control. The test was conducted in the SCJEL_btfm_ (E113.9, N35.3), and the thickness and humidity of the solid medium were 1.5 cm and 70%, respectively. Lipid yield of *P. chrysosporium* was determined during cultivation. The lyophilized fungal mattress and biomass of *P. chrysosporium* were homogenized using a mortar and pestle and then solvent extraction was performed in accordance with the modified Bligh and Dyer method to obtain the TLE [36].

### Lipid component identification by metabolomic analysis

The total lipid extract (TLE, 30 ± 1 mg) was obtained using 0.4 mL n-hexane and 20 μL L-2-chlorophenylalanine (1 mg/mL stock in dH_2_O) and placed in 2 mL Eppendorf tubes. After vortex mixing (30 s) and centrifugation (15 min) at 12,000 rpm and 4 °C, the supernatant (0.4 mL) was transferred into a fresh gas chromatography–mass spectrometry (GC–MS) glass vial and dried completely in a vacuum concentrator at 25 °C. The sample was incubated with 70 μL of methoxy amination hydrochloride (20 mg/mL in pyridine) for 30 min at 80 °C and then with 90 μL of the BSTFA reagent (Regis Technologies, Inc., Morton Grove, IL, USA) for 1.5 h at 70 °C. After these treatments, all samples were analyzed with an Agilent 7890 GC system coupled with a Pegasus HT time-of-flight mass spectrometer (TOFMS). The system utilizes a DB-5MS capillary column coated with 5% diphenyl cross-linked with 95% dimethylpolysiloxane (30 m × 250 μm inner diameter, 0.25 μm film thickness; J&W Scientific, Folsom, CA, USA). An aliquot (1 μL) of the analyte was injected in splitless mode. Helium was employed as the carrier gas, and its flow rate through the column was 1 mL/min. The initial temperature was maintained at 50 °C for 1 min, then raised to 310 °C at a rate of 10 °C/min, and maintained for 8 min at 310 °C. The injection, transfer line, and ion source temperatures were 280 °C, 270 °C, and 220 °C, respectively. The energy was maintained at − 70 eV in electron impact mode. MS data were acquired in the full-scan mode with an m/z range of 50–500 at a rate of 20 spectra per second after a solvent delay of 6.17 min. Chroma TOF 4.3X software of LECO Corporation and LECO-Fiehn Rtx5 database were used for data processing and annotation, including baseline calibration and filtering, peak alignment, deconvolution analysis, raw peak exacting, peak identification, and peak area integration [37]. The mass spectrum match and retention index match were considered during metabolite identification.

### Fluorescence and laser scanning confocal microscopy (LSCM) observation

The cultures from the SC and SSC of *P. chrysosporium* were observed by fluorescence microscopy (Leica DM2500, Germany) and LSCM (Zeiss LSM 800, Germany). Before observation, the cultures were dyed using Nile red (0.5 g/L) and Calcofluor fluorescent stains (mixture of 1.0 g/L Calcofluor white and 0.5 g/L Evans blue) to highlight the cell wall. Subsequently, the intracellular lipid content of the fungal cells was estimated.

### RNA sequencing and KEGG analysis

Four samples including the cultures from SC (Ck1 on day 4; Ck2 on day 8) and SSC (T1 on day 4; T2 on day 9) were prepared. Sample Ck1 was used as the inoculum in SSC, and Ck1 can be seen as the cultures of SSC on day 0 (T0). These samples were immediately frozen in liquid nitrogen and then stored at − 80 °C until RNA isolation. Total RNA was extracted using a Trizol reagent according to the manufacturer’s protocol (Invitrogen, China) and then treated with DNase to remove DNA contamination. The yield and purity of RNA sample were checked using a NanoDrop™ 2000 spectrophotometer (Thermo Scientific, USA) at 260 nm and 280 nm. The integrity of all RNA samples was assessed by 1.0% agarose gel. The mRNA from total RNA was isolated and enriched using oligo (dT) magnetic beads (Illumina, CA, USA). Subsequently, mRNA was fragmented to short fragments to be used as templates for random hexamer-primed synthesis of first-strand cDNA by fragmentation buffer. Second-strand cDNA was synthesized using buffer, dNTPs, RNase H, and DNA polymerase I. A paired-end cDNA library was synthesized using a Genomic Sample Preparation Kit (Illumina, CA, USA) according to the manufacturer’s instructions. Short fragments were purified with a QiaQuick polymerase chain reaction (PCR) extraction kit (Qiagen, Germany) and eluted in 10 µL of elution buffer. An Agilent 2100 Bioanalyzer (Agilent Technologies, Santa Clara, CA, USA) and ABI Step One Plus Real-Time PCR System (Applied Biosystems, CA, USA) were used to examine the quality and quantify the sample library [38]. Finally, cDNA libraries were sequenced on an Illumina HiSeq™ 2500 (Novogene, Beijing, China), and the data obtained have been submitted into the NCBI Sequence Read Archive under the accession number of PRJNA495103.

Raw reads were cleaned by removing adapter sequences, empty reads, and low-quality sequences. For annotation analysis, unigenes were BLASTX-searched against five databases, namely the National Center for Biotechnology Information (NCBI) nonredundant (NR) protein sequence database, the NCBI NR nucleotide sequence (NT) database, Kyoto Encyclopedia of Genes and Genomes (KEGG) orthology database, Swissprot, and PFAM database, using a cut-off E-value of 10^−5^. To eliminate the influence of different gene lengths and sequence discrepancies on expression calculations, gene expression levels based on read counts obtained by RSEM (version v1.2.15) were normalized using FPKM (fragments per kilo bases per million fragments) transformation [39]. The calculated gene expression levels were used for direct comparison among samples. Expression values were standardized across the dataset to enable the data from different genes to be combined.

Using the R package DEGseq, differentially expressed genes (DEGs) were identified with a random sampling model on the basis of the read count for each gene at different developmental stages. False discovery rate ≤ 0.05 and absolute value of |log2Ratio|≥1 were set as the threshold for significance of gene expression differences between adjacent samples. The KEGG database was used to assign and predict putative functions and pathways associated with the assembled sequences [40].

### Statistical analytical methods

Statistical analysis was conducted in SPSS19.0 for Windows. All data were presented as mean ± standard error of the mean and evaluated using one-way ANOVA of the three biological replicates followed by the least significant difference test, with *p* < 0.01 and *p* < 0.05.

## Results and discussion

### Effect of different cultivation strategies on lipid production

**Fig. 1:**
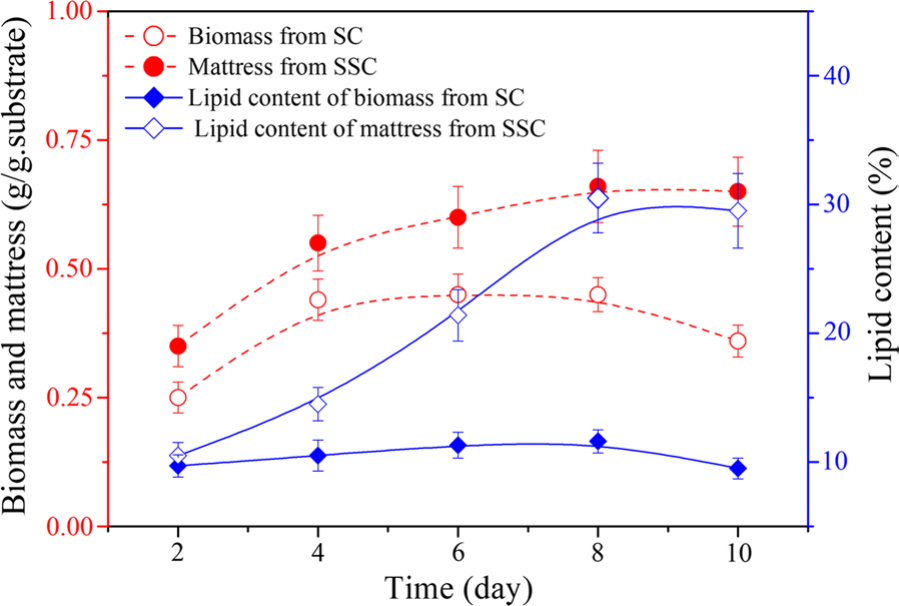
Variations in biomass or mattress yield and lipid content with time during SC and SSC

Figure 1 shows that the different cultivation strategies significantly influenced the biomass and lipid production. The maximum biomass from SC was achieved on day 6 at 0.470 g/g substrate. Meanwhile, SSC resulted in the formation of a fungal mattress on the surface of solid medium, and the fungal mattress reached 0.66 g/g substrate on day 8, although the cultivation period was longer than that in the SC. Notably, the heavier mattress in the SSC was partly caused by the fungal metabolites and retained substrates, which were difficult to remove completely and hence included in the mattress. In addition, the fungal mattress had a high lipid content of 30.5%, which was 2.44 times the maximum lipid content (11.3–11.5%) of the biomass obtained by SC on day 6–8. By calculation, the SSC lipid yield, expressed as the conversion efficiency of substrates to lipid, reached 0.201 g/g substrate, which was significantly higher than that of SC (0.06 g/g substrate). This result indicates that SSC is conducive for lipid production from *P. chrysosporium.* In submerged cultures supplied with olive oil, *P. chrysosporium* mycelium was reported to exhibit enriched phospholipid and fatty acid contents relative to those in lipid-free medium [41, 42]. However, to the best of our knowledge, limited information is available on the overproduction of fungal lipids by SSC.

### Optimization of cultivation factors

**Fig. 2:**
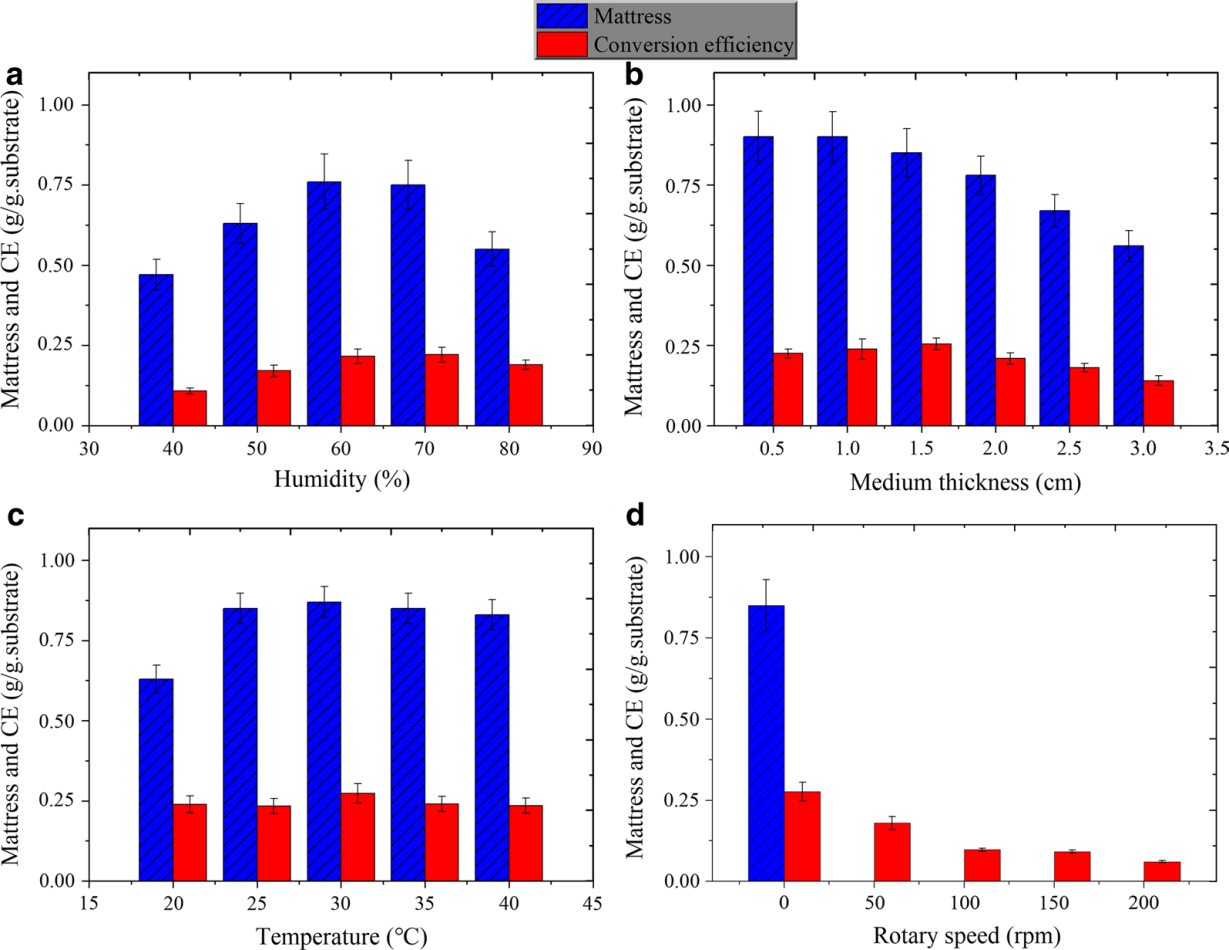
Effects of humidity, solid medium thickness, temperature, and rotary speed on the biomass and lipid conversion efficiency. **a** Humidity, **b** solid medium thickness, **c** temperature, and **d** rotary speed

Medium humidity significantly influences lipid yield. In a humidity test, 60–70% of the initial humidity of the solid medium was suitable for lipid production (Fig. 2a). The lipid conversion efficiency reached 0.216–0.221 g/g substrate. A very high (≥ 80) or low humidity (≤ 50%) led to the decrease in mattress weight and lipid yields.

Results of medium thickness test showed that the lipid yield decreased with solid medium thickness exceeding 2.0 cm. This is because the underlying medium cannot be fully utilized. At the bottom, the fungal inoculum did not develop well due to the lack of oxygen, which led to the lighter mattress in the solid-state cultivation (Fig. 2b). Moreover, a very thin medium indicates the low production capacity of the reactor. Therefore, 1.5 cm of medium thickness was suggested for lipid production of *P. chrysosporium* by SSC.

Temperature is an important factor influencing fungal growth and metabolism. A too high (> 45 °C) or too low (< 15 °C) temperature is not suitable for the growth of *P. chrysosporium* used. Figure 2c clearly shows that the fungus propagated more effectively under the temperatures of 20–40 °C, although the mattress obtained at 20 °C was significantly lighter than those achieved at other temperatures. Interestingly, on day 10, differences in lipid yield at different temperatures were insignificant (*p* > 0.05), although a maximum lipid conversion efficiency (0.273 g/g substrate) was obtained at 30 °C. Therefore, the temperature did not significantly influence the lipid yield in the range of 20–40 °C, probably because low temperature, although unsuitable for fungal growth, increased the lipid content by inducing lipid synthesis [43].

During the solid cultivation of the bioreactor, shaking or stirring can improve the mass transfer efficiency of the substrate by enhancing contact of the fungus with air and substrates [44, 45]. In the rotary speed test, the mattress was not obtained because the fungal hyphae and substrates were mixed together by shaking. Figure 2d shows that shaking significantly reduced lipid conversion. Thus, SSC was considered as the suitable strategy for lipid accumulation in *P. chrysosporium* cells, and under optimized conditions, the lipid conversion efficiency reached 0.277 g/g substrate on day 9. Productivity also reached 69.5 g/m^2^/day based on the 6.5 cm diameter of the glass bottle used in this work.

### Lipid component analysis based on GC-TOFMS

**Fig. 3:**
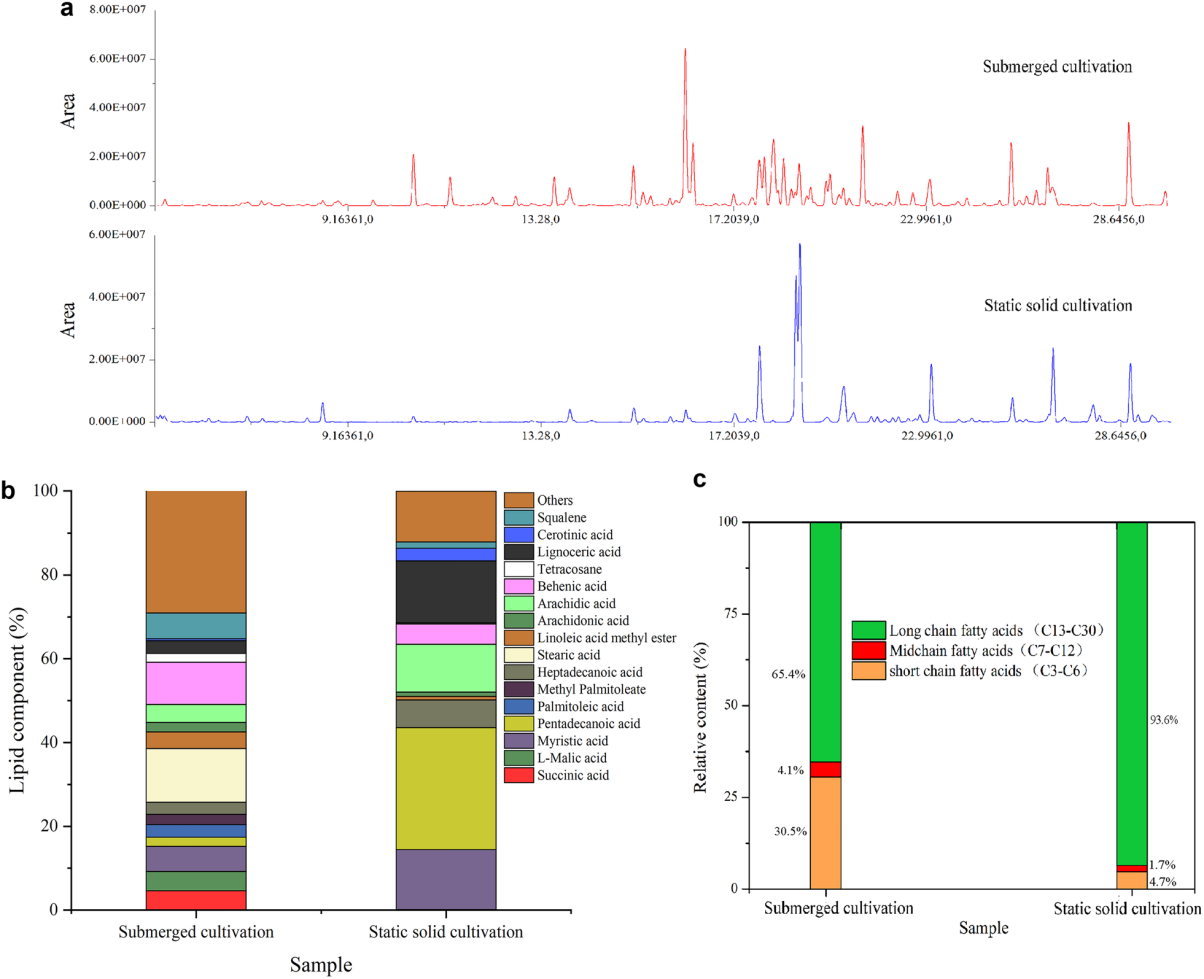
Lipid component analysis based on GC-TOFMS. **a** Total ion currents of GC-TOFMS of TLEs from SC and SSC; **b** component comparison of fatty acids and their esters produced by SC and SSC; and **c** relative contents of long-chain, midchain, and short-chain fatty acids in the lipids obtained by SC and SSC

By lipid-targeted metabolomic analysis, 97 fatty acids and their esters or other derivatives were identified in the TLE samples. The total ion currents of the GC-TOFMS of TLEs from SC and SSC (Fig. 3a) showed different cultivations resulting in the difference of fatty acids in both kind and content. Compared with SC, the lipid component in SSC was relatively simple, and approximately 34 kinds of fatty acids were absent. Figure 3b reveals that the dominant fatty acids produced by SC were stearic acid (12.8%), behenic acid (10.1%), and squalene (6.1%), and those obtained by SSC included pentadecanoic acid (29.1%), myristic acid (14.4%), lignoceric acid (14.8%), and arachidic acid (11.5%). Moreover, more than 90% of the fatty acids produced by SSC were saturated. A high proportion of saturated lipids are suitable for biodiesel production because the higher the unsaturated methyl ester content, the higher the biodiesel oxidation potential, which may incur additional problems in fuel polymerization during combustion [46, 47]. Furthermore, Fig. 3c shows that the lipids produced by SSC contained more long-chain fatty acids (93.6%) than those by SC (65.4%).

### Morphology of *P. chrysosporium* cultures

**Fig. 4:**
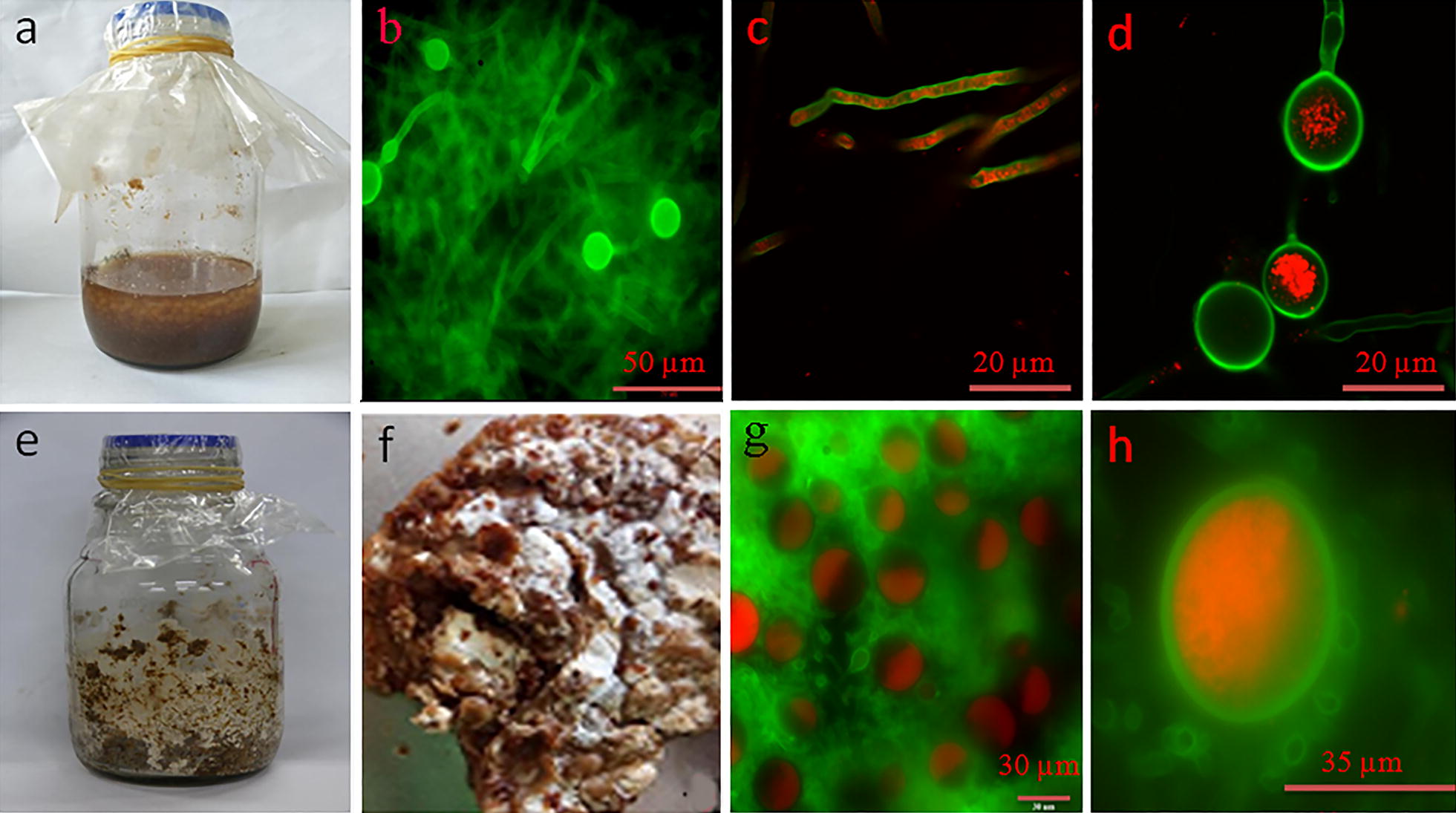
Microscopic observation of the morphological changes of *P. chrysosporium* during SC and SSC. **a** SC; **b** hyphae and chlamydospore-like cells under fluorescence microscopy; **c** hyphae and **d** chlamydospore-like cells under LSCM; **e** SSC; **f** fungal mattress; **g** chlamydospores under LSCM and **h** chlamydospores filled with lipids stained with Nile red

The mechanism by which different cultivation strategies influence lipid production remains unclear. Microscopic observation reveals that the cultures obtained by SC were mainly composed of hyphae and a few chlamydospore-like cells (Figs. 4a, b). Under LSCM, the normal hypha is filamentous, whereas the chlamydospore-like cell is a global or elliptic reproductive cell with a thick cell wall (Figs. 4c, d). However, SSC resulted in the formation of a large fungal mattress containing numerous chlamydospores (Fig. 4e–g).

The morphological changes during the cultivation of *P. chrysosporium* are interesting. The conidium, the fungal propagule, generally appears on the surface of solid media. After germination, the conidium develops into a hypha, and as a filamentous fungus, *P. chrysosporium* commonly forms a hypha. Wang et al. [48] reported that the chlamydospore-like cell of *P. chrysosporium* has an average diameter of 15 μm, and the specific cell may be an intermediate structure between the hypha and chlamydospore of the fungi. Such observation demonstrates the characteristics of metabolically active entities, such as an enzyme reservoir [49]. The “true” chlamydospore of *P. chrysosporium*, typically having a diameter of up to 60 μm [50], has been rarely explored. Notably, the chlamydospores with an average diameter of 35 μm contained more lipids than those in the hypha and chlamydospore-like cell by LSCM (Fig. 4h). This phenomenon explains why the fungal mattress obtained by SSC contained more lipids than that in SC. During the formation of chlamydospores of *Pullularia pullulans*, an increase in the proportion of long-chain saturated fatty acids in total lipids was found [51]. Generally, chlamydospores are resistance forms induced by the hostile environmental conditions such as low temperature [52, 53], and thus they accumulate large amounts of fatty acids and have their fatty acids in a long-chain saturated state to serve as a reserve nutrient. This finding not only explains why low temperature is helpful for lipid accumulation, but also clarifies why chlamydospores in SSC contain more long-chain fatty acids.

### RNA sequencing datasets and analysis

A summary of RNA-Seq data quantity is shown in Additional file 1: Table S1. After the quality check, the sequencing of 12 cDNA samples of *P. chrysosporium* correspondingly yielded 39,796,010–49,017,238 clean sequences. Good quality scores of the sequences were calculated, and the Q20 and Q30 percentages were higher than 99.42% and 93.21%, respectively, which showed that the sequencing results were sufficient and reliable [54].

**Fig. 5:**
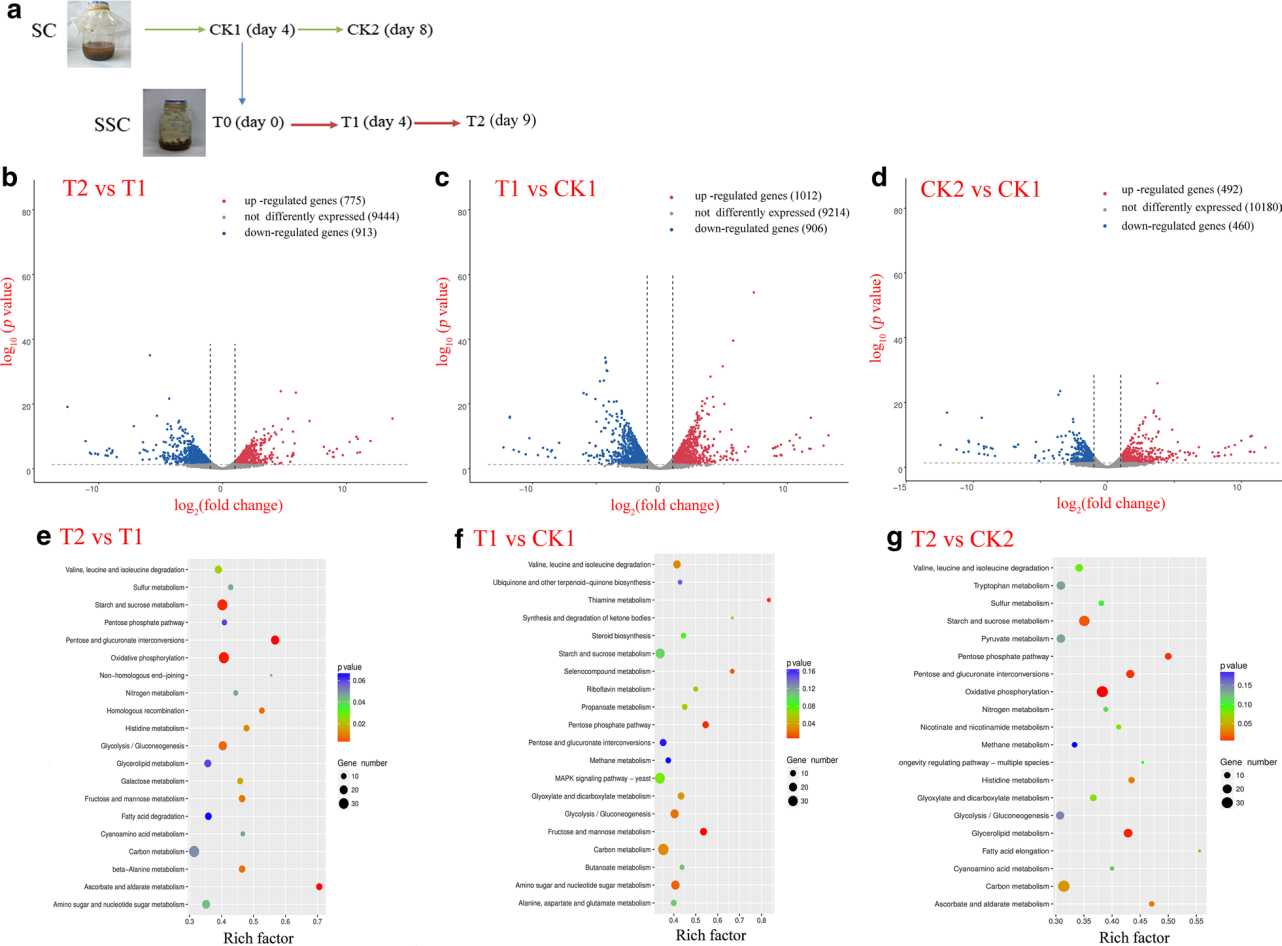
The sampling design and RNA sequencing analysis. **a** The sampling design for RNA sequencing; **b**–**d** the scatter-plots of differentially expressed genes from transcriptomes of samples; **e**–**g** KEGG pathway enrichment analysis of DEGs

To understand better the variety of genes in *P. chrysosporium* under different cultivation conditions, the DEGs in different samples were visualized by calculating the FPKM value of genes. Figure 5a shows the sampling design for RNA sequencing, and 15.2% genes in T2 versus T1 and 17.2% genes in T1 versus Ck1 were significantly different expressed during SSC (Fig. 5b, c). In SC, the up-regulated and down-regulated genes in Ck2 versus Ck1 were 492 and 460, respectively, which is only 8.6% of the total genes (Fig. 5d). This fact indicates the changes of gene expression in SC are less than those in SSC.

**Fig. 6:**
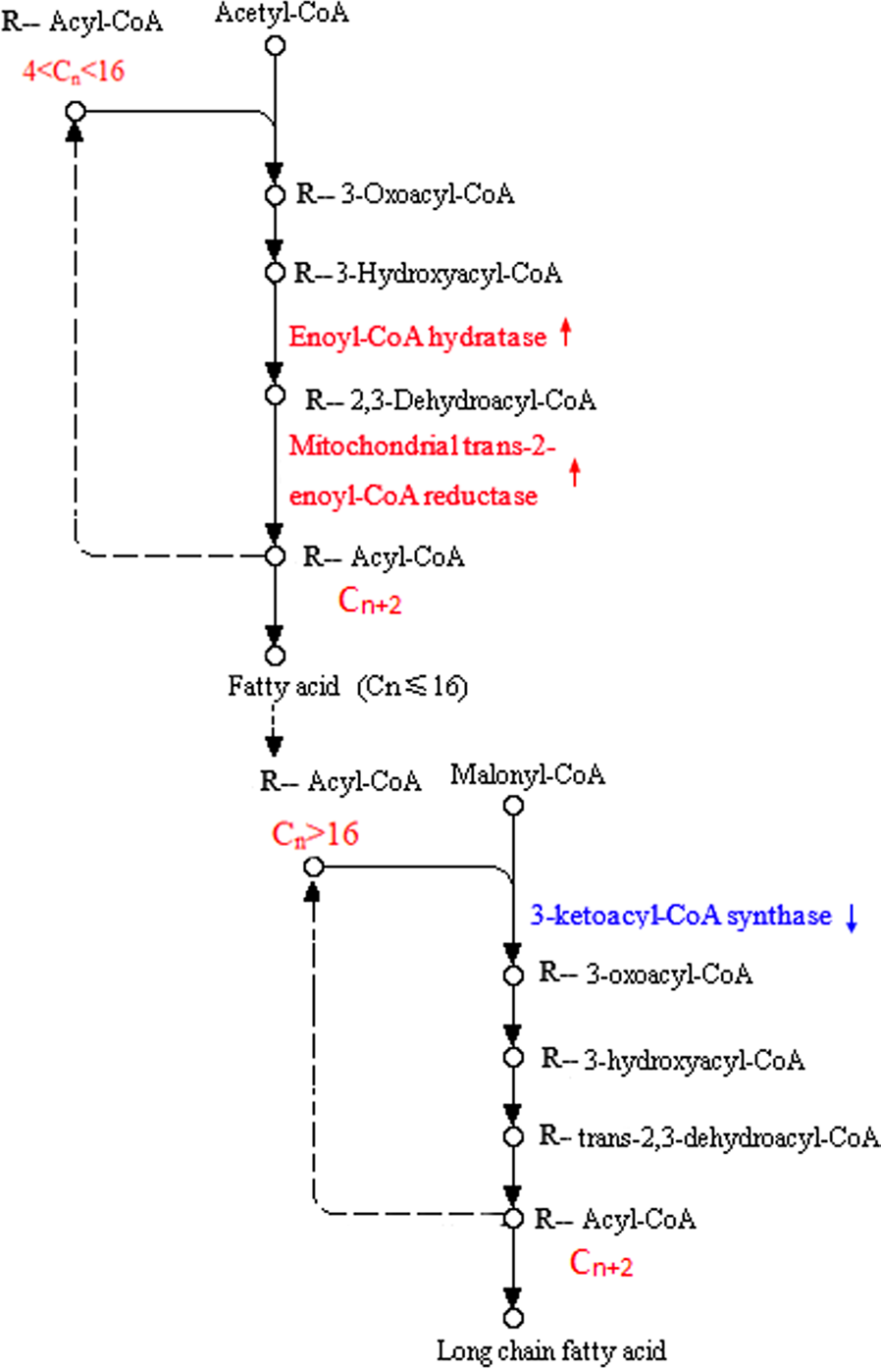
A simplified fatty acid elongation pathway in *P. chrysosporium*. The red arrow represents the significantly up-regulated DEG and the blue arrow represents the significantly down-regulated DEG. Fold change is the change of gene expression between samples

KEGG pathway-based analysis not only elucidates the biological functions of genes, but also further identifies significantly enriched metabolic pathways or signal transduction pathways in DEGs against the whole genome background [55]. After KEGG pathway analysis, the DEGs were generally placed into different pathways. Figure 5e shows that the DEGs involved in fatty acid synthesis were not significantly changed into samples T2 versus T1. However, 14 DEGs involved in fatty acid degradation were enriched (*p *= 0.06), especially the long-chain acyl-CoA synthetase [EC: 6.2.1.3] which catalyzes long-chain fatty acid to form long-chain acyl-CoA was significantly down-regulated (Additional file 1: Fig. S1), suggesting that the fungus accumulated lipid by reducing fatty acid consumption at its decline phase during SSC. Figure 5f, g shows that fatty acid metabolism between samples T1 and CK1 in rapid growth phase (on day 4 for both SC and SSC) has no significant difference. However, the DEGs involved in glycerolipid metabolism (18 DEGs) and fatty acid elongation (5 DEGs) were enriched into samples T2 versus Ck2. Fatty acid biosynthesis is closely related to glycerolipid metabolism and fatty acid elongation. Glycerolipid metabolism can consume or produce large amounts of fatty acids, and fatty acid elongation is beneficial to synthesize long-chain fatty acids [56, 57]. At the late stage of cultivations (on day 8 for SC and day 9 for SSC), the significant up-regulation of fabG (3-oxoacyl-[acyl-carrier protein] reductase, EC: 1.1.1.100) helps fatty acid biosynthesis (Additional file 1: Fig. S2), suggesting that the chlamydospores cultivated by SSC still possess the higher ability to synthesize fatty acids compared to the hyphae cultivated by SC even at the fungal decline phase. Figure 6 exhibits a simplified fatty acid elongation pathway in *P. chrysosporium*. The genes coding enoyl-CoA hydratase (EC: 4.2.1.17) and mitochondrial trans-2-enoyl-CoA reductase (EC: 1.3.1.38) were significantly up-regulated, and the up-regulated genes explain why the saturated long-chain fatty acids are synthesized in large quantities in chlamydospores. The down-regulated gene coding 3-ketoacyl-CoA synthase (EC: 2.3.1.199) reveals why pentadecanoic acid (C15) and myristic acid (C14) accounting for 45% of total lipids were accumulated. Therefore, the DEGs found in the fatty acid metabolism pathway present an answer to the quantity and types of fatty acids accumulated in chlamydospores.

### Feasibility analysis in conversion efficiency

At present, microbial oils are mostly produced by SC, and few cases of lipid production by solid-state fermentation have been successful. Table 2 shows that lipid yield of *P. chrysosporium* by SSC almost reached the maximum conversion efficiency of yeast reported in literature and had an obvious advantage over those of bacteria, other fungi, and heterologous microalgae by SC. The theoretical lipid conversion by microorganisms is about 0.310 g/g when glucose is used as substrate [26]. Therefore, 0.277 g/g substrate of lipid yield obtained in this work proved that SSC is a promising and feasible method for microbial lipid production. Notably, autotrophic microalgae grow phototrophically, and their lipid conversion efficiencies are not discussed in this paper because of difficulty in evaluating the solar-to-biomass conversion. The reasons behind the increased conversion efficiency obtained are as follows: (i) The chlamydospore of *P. chrysosporium* is a special cell structure for storing lipids, and their presence in the fungal mattress improved the lipid yield. (ii) Moreover, in the SC of yeast, bacteria, filamentous fungi, and microalgae, the biomass yields were lower than those under SSC, although the lipid content reached 60–80%. However, in this experiment, the mattress accounted for up to 85% of the substrates by weight. Clearly, the fungal mattress contained not only biomass but also the retained wheat bran and glucose, which were not consumed completely. Table 1 indicates that there are also lipids in wheat bran, and this portion of the lipids from the unused substrates may have been extracted and calculated into the fungal lipids.

**Table 2:** Comparison of lipid conversion efficiency among different oleaginous microorganisms

Microorganisms	Cultivation method	Conversion efficiency (g/g substrate)	References
*Candida* 107 (Y)	SC	0.052 g/g glucose	[58]
*Yarrowia lipolytica* (Y)	SC	≤ 0.270 g/g.glucose	[8, 59]
*Cryptococcus curvatus* (Y)	SC	0.11–0.21 g/g.sugar^b^	[60]
*Trichosporon cutaneum* (Y)	SC	0.12 g/g galacturonate	[26]
*Rhodotorula glacialis* (Y)	SC	0.16 g/g glucose	[61]
*Debaryomyces etchellsii* (Y)	SC	≤ 0.031 g/g glucose	[62]
*Rhodococcus erythropolis* (B)	SC	0.061 g/g glucose	[63]
*Bacterium* GK12 (B)^a^	SC	< 0.098 g/g gluconate	[64]
*Rhodococcus opacus* (B)	SC	0.041 g/g lignin	[65]
*Bacillus subtilis* (B)	SC	0.045 g/g reducing sugar	[66]
*Escherichia coli* (B)	SC	< 0.034 g/g ethanol	[67]
*Mortierella isabellina* (F)	SC	0.17 g/g glucose	[68]
*Cunninghamella echinulata* (F)	SC	0.21 g/g tomato waste hydrolysate	[69]
*Mucor* sp. (F)	SC	0.058 g/g glucose	[70]
*Mortierella ramanniana* (F)	SC	0.15 g/g glucose	[71]
*Chlorellazofi ngiensis* (HM)	SC	0.136 g/g glucose	[72]
*Chlorella sorokiniana* (HM)	SC	0.19 g/g glucose	[73]
*Aspergillus oryzae* A-4 (F)	SSF	0.0366 g/g dry substrate	[74]
*Aspergillus tubingensis* TSIP9 (F)	SSF	0.047 g/g dry substrate	[75]
*Phanerochaete chrysosporium* [F]	SSC	0.277 g/g WCG	This work

^a^A novel species affiliated with the family Erysipelotrichaceae in the phylum Firmicutes

^b^Sugar includes arabinose, galacturonate, glucose, and beet pulp hydrolysates

Y, B, F, and HM represent yeast, bacterium, fungus, and heterotrophic microalgae, respectively

SC means submerged cultivation, whereas SSC stands for static solid cultivation

WCG refers to the mixture of wheat bran, corn straw, and glucose

### Engineering viability and cost–benefit analysis

**Fig. 7:**
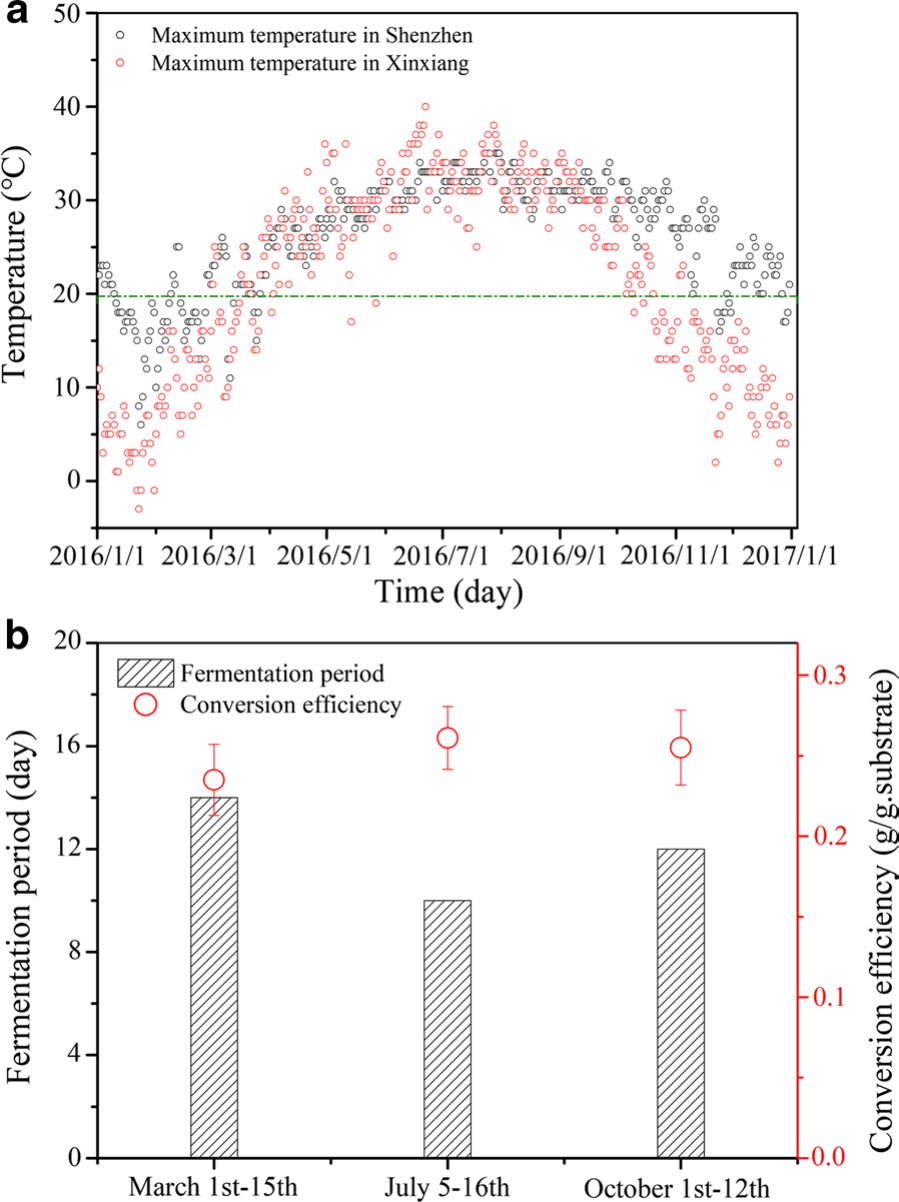
Annual temperature variations in Xinxiang and Shenzhen in 2016 and the lipid cultivation test without temperature control in Xinxiang, China. **a** Temperature variations in Xinxiang and Shenzhen in 2016. **b** Conversion efficiency and fermentation period of the lipid production test conducted in March, July, and October in Xinxiang in 2016

A crucial challenge that needs to be considered for microbial lipid production is cost viability in the industrial scale. The production costs mainly include substrate cost, reactor operational cost, and extraction cost of microbial lipids. During fermentation, the energy and water consumption as well as the power, steam, and cooling water for temperature control, aeration, and stirring accounted for most of the total reactor operational cost (> 90%). However, the optimization results (Fig. 2c, d) presented in this work showed that the lipid fermentation of *P. chrysosporium* by SSC is promising in diminishing the reactor operational cost. In comparison with SC, neither stirring nor aeration is necessary in SSC. Additionally, *P. chrysosporium* can grow and produce lipids in a wide temperature range (20–40 °C), which offers the fungal fermentation an obvious advantage in temperature control. For example, in Xinxiang City of north China, the number of days with temperatures exceeding 20 °C is 210 and mainly distributed from March to November (Fig. 7a). Meanwhile, the number of days with such temperature in the cities of south China, such as Shenzhen, is 293. This result means that SSC can be conducted without temperature control except in January and February. Certainly, SSC is also suitable in the tropical and subtropical countries or areas in Asia, Africa, and America.

To validate the feasibility of lipid production without temperature control, we conducted a robust cultivation test at room temperature in the SCJEL_btfm_ in March, July, and October. The experiments obtained conversion efficiencies ranging from 0.245 g/g substrate to 0.261 g/g substrate in different seasons, suggesting the feasibility of SSC in microbial lipid production (Fig. 7b). In March, the fungal growth under a wide temperature variation (10–28 °C) due to day and night alternation was not as rapid as that under thermostat conditions. Hence, the cultivation period was extended to 14 days. The extension of the cultivation period did not distinctly increase the costs because stirring, aeration, and temperature control were not required. The conversion efficiency of 0.245 g/g substrate achieved in March implied that decreased temperature and prolonged cultivation period did not significantly affect the lipid accumulation.

A simple reactor without stirring, aeration, and temperature control systems conserves heat energy, power, and water consumption and instrument and meter use. Table 3 shows the cost reduction of the fungal lipid production by SSC relative to that in the SC of the oleaginous yeast *Apiotrichum curvatum*, which offers a higher lipid productivity (0.995 g/L/h) as reported by Tsouko et al. [76]. For instance, in a 10-ton fermenter, approximately 1.44 tons of TLE was produced by *A. curvatum* after 6 days’ cultivation, and 1.37 tons of biodiesel worth 6156 RMB was obtained at a 95% biodiesel catalysis efficiency and 4500 RMB worth of biodiesel [22]. However, a 30 kW/h air compressor for aeration and a 22 kW/h stirrer installed on the fermenter obtained electricity costs of 2712.6 and 1995.8 RMB, respectively. During SC, the temperature usually rises after start-up due to the heat generated by microbial metabolism. Approximately 648 tons of cooling water (costing 1814.4 RMB) at a flow rate of 4.5 tons/h was used to control the fermenter temperature. Clearly, even if the heat energy consumed during reactor start-up and the losses under instrument and equipment usage were not accounted for, the consumed power and water already reached 6522.84 RMB (approximately 959.24$ based on a 6.8 exchange rate) for the 6-day operation of a 10-ton fermenter. Such cost already surpassed value of the obtained biodiesel in recent years. However, SSC can diminish these reactor operational costs, including water and power expenses, because temperature, aeration, and stirring control are not needed. Thus, SSC is a robust and cost-saving cultivation strategy for the *P. chrysosporiu*m used in industrial-scale lipid production.

**Table 3:** Water and power costs in SC and SSC

Cultivation strategy	Submerged cultivation^a^	Static solid cultivation
Power for aeration (RMB)	2712.6	0
Power for stirrer (RMB)	1995.8	0
Cooling water (RMB)	1814.4	0
Power for heater (RMB)	N/A	0
Equipment depreciation	N/A	N/A
Total cost (RMB)	6522.8	0
Biodiesel value (RMB)	6120^b^	6120

N/A, not available

^a^A 10-ton liquid fermenter is taken as an example. The power parameters for the air compressor (30 kW/h) and stirrer (22 kW/h) and the flow rate of cooling water (4.5 tons/h) were provided by the Fermentation Factory of Tuoxin Group, Xinxiang, China. The water and electricity expenses for industrial use were 2.80 RMB/ton and 0.63 RMB/kWh, respectively

^b^ Lipid production was calculated based on a lipid productivity of 0.995 g/L/h. Approximately 1.43 tons of lipids and 1.36 tons of biodiesel were produced after 6 days’ fermentation under a 95% biodiesel catalysis efficiency. The biodiesel value was evaluated based on a price of 4500 RMB (The renminbi, the official currency of the People’s Republic of China)/ton

Certainly, SSC also holds several limitations. It is only suitable for lipid production by filamentous fungi because bacteria, yeast, and microalgae cannot form mattresses on solid medium surfaces. A space larger than usual is also needed for scale-up SSC because of the thinner solid medium used (1.5 cm). A feasible reactor and process design should be developed further because of the few successful SSC cases available for reference. Therefore, some problems still need to be resolved before industrial lipid production by the fungus is realized. Nevertheless, SSC is a promising strategy for the fungus with temperature robustness as it conserves almost the entire reactor operational cost of lipid production.

## Conclusions

This study first confirmed that producing microbial lipids by a robust and cost-saving solid fermentation strategy is feasible and promising. *P. chrysosporiu*m can produce lipids via SSC with a lipid conversion efficiency of up to 0.277 g/g substrate under optimized cultivation parameters. LSCM technology revealed the significant morphological changes of *P. chrysosporiu*m during the cultivations. The presence of chlamydospores containing more lipids than the hyphae and chlamydospore-like cells explains why the lipid yield was higher in SSC than in SC. RNA sequencing revealed that the chlamydospores stored lipid by reducing fatty acid consumption. In addition, compared to SC, the genes coding the enzymes related to fatty acid metabolism including fabG, enoyl-CoA hydratase, 3-ketoacyl-CoA synthase and so forth were significantly changed, which uncovered the reason for the appearance of abundant saturated long-chain fatty acids in chlamydospores during SSC. More importantly, engineering viability and cost–benefit analysis showed that SSC conserved almost all of the reactor operational cost for lipid production unlike SC, which requires water- and energy-consuming equipment and aeration, stirring, and temperature control devices. Thus, SSC is promising for the fungus with temperature robustness in industrial lipid production.

## Additional file

**Additional file 1.** Additional figures and table.

## Data Availability

Additional file 1 provides additional data.
