# Supplementary material for: Robust and cost-saving static solid cultivation method for lipid production using the chlamydospores of Phanerochaete chrysosporium
Source: Biotechnol Biofuels. 2019 May 20;12:123. doi: 10.1186/s13068-019-1464-1 (PMC6528298; doi:10.1186/s13068-019-1464-1)
Supplement: Supplementary file 1 — Additional file 1. Additional figures and table. [file 13068_2019_1464_MOESM1_ESM.docx]

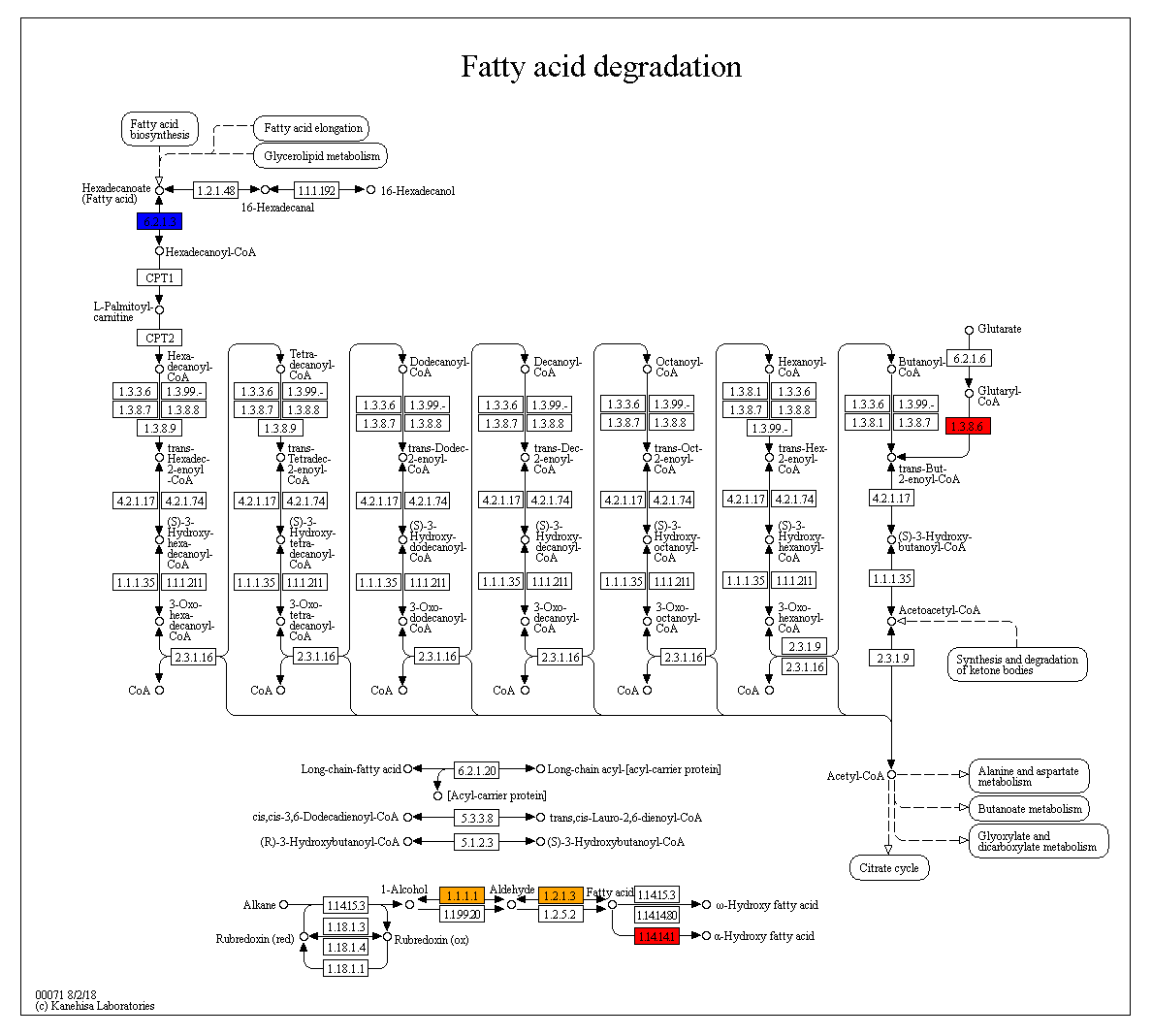


**Fig. S1** The differentially expressed genes (DEGs) involved in fatty acid degradation in samples T2 versus T1 by KEGG analysis. The red box represents the significantly up-regulated DEG; the blue box represents the significantly down-regulated DEG, and the orange box represents that both the significantly up-regulated and significantly down-regulated DEG exist in this node.

**
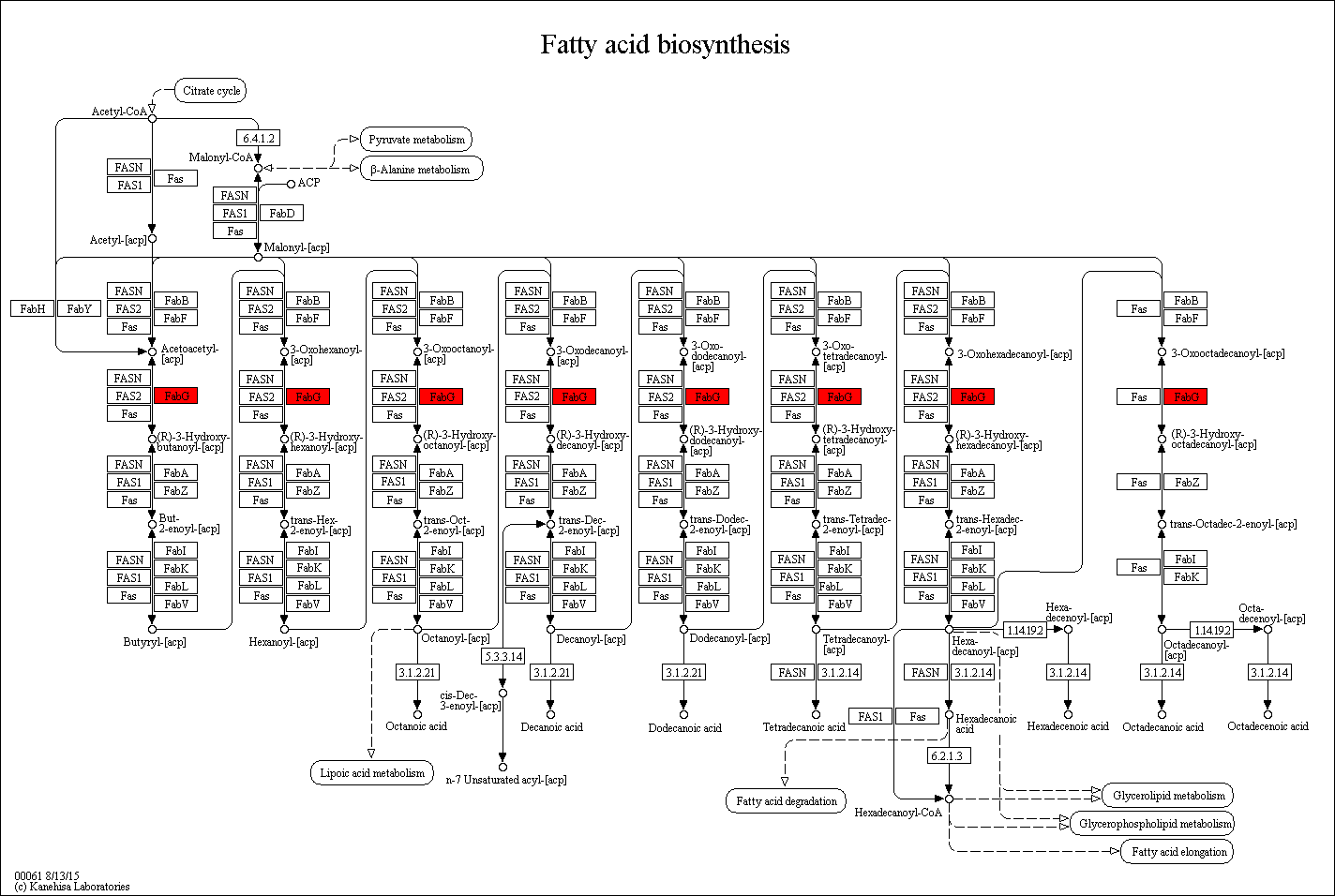
**

**Fig. S2** The DEG involved in fatty acid biosynthesis in samples T2 versus CK2 by KEGG analysis. The red box represents the significantly up-regulated DEG.

Table S1 Summary of RNA sequencing data quantity

| **Sample** | **Valid data**  **(reads)** | **Valid data**  **(bases)** | **Valid ratio**  **(%)** | | **Q20**  **（%）** | **Q30**  **（%）** | **GC content**  **（%）** |
| --- | --- | --- | --- | --- | --- | --- | --- |
| CK1a* | 39796010 | 5.97G | 99.59 | 99.49 | | 93.67 | 58.5 |
| CK1b | 47246210 | 7.09G | 99.60 | 99.42 | | 93.37 | 59.0 |
| CK1c | 45736944 | 6.86G | 99.62 | 99.42 | | 93.21 | 59.0 |
| CK2a | 44224156 | 6.63G | 98.63 | 99.72 | | 97.00 | 59.0 |
| CK2b | 48724038 | 7.31G | 99.42 | 99.45 | | 93.61 | 59.5 |
| CK2c | 45825160 | 6.87G | 99.58 | 99.54 | | 94.02 | 58.5 |
| T1a | 47286592 | 7.09G | 99.53 | 99.53 | | 93.96 | 59.5 |
| T1b | 47610400 | 7.14G | 99.50 | 99.55 | | 94.21 | 59.5 |
| T1c | 42237572 | 6.34G | 99.40 | 99.48 | | 93.45 | 59.5 |
| T2a | 45193228 | 6.78G | 99.30 | 99.56 | | 94.02 | 58.5 |
| T2b | 49017238 | 7.35G | 99.55 | 99.53 | | 93.88 | 58.5 |
| T2c | 43529532 | 6.53G | 99.31 | 99.48 | | 93.77 | 58.5 |

* , Letters a, b and c represent three repeats of the same sample.
